# Exogenous Visual Orienting Is Associated with Specific Neurotransmitter Genetic Markers: A Population-Based Genetic Association Study

**DOI:** 10.1371/journal.pone.0030731

**Published:** 2012-02-14

**Authors:** Rebecca A. Lundwall, Dong-Chuan Guo, James L. Dannemiller

**Affiliations:** 1 Psychology Department, Rice University, Houston, Texas, United States of America; 2 Department of Internal Medicine, University of Texas Health Science Center, Houston, Texas, United States of America; Harvard University, United States of America

## Abstract

**Background:**

Currently, there is a sense that the spatial orienting of attention is related to genotypic variations in cholinergic genes but not to variations in dopaminergic genes. However, reexamination of associations with both cholinergic and dopaminergic genes is warranted because previous studies used endogenous rather than exogenous cues and costs and benefits were not analyzed separately. Examining costs (increases in response time following an invalid pre-cue) and benefits (decreases in response time following a valid pre-cue) separately could be important if dopaminergic genes (implicated in disorders such as attention deficit disorder) independently influence the different processes of orienting (e.g., disengage, move, engage).

**Methodology/Principal Findings:**

We tested normal subjects (N = 161) between 18 and 61 years. Participants completed a computer task in which pre-cues preceded the presence of a target. Subjects responded (with a key press) to the location of the target (right versus left of fixation). The cues could be valid (i.e., appear where the target would appear) or invalid (appear contralateral to where the target would appear). DNA sequencing assays were performed on buccal cells to genotype known genetic markers and these were examined for association with task scores. Here we show significant associations between visual orienting and genetic markers (on *COMT*, *DAT1*, and *APOE*; *R^2^s* from 4% to 9%).

**Conclusions/Significance:**

One measure in particular – the response time cost of a single dim, invalid cue – was associated with dopaminergic markers on *COMT* and *DAT1*. Additionally, variations of *APOE* genotypes based on the ε2/ε3/ε4 alleles were also associated with response time differences produced by simultaneous cues with unequal luminances. We conclude that individual differences in visual orienting are related to several dopaminergic markers as well as to a cholinergic marker. These results challenge the view that orienting is not associated with genotypic variation in dopaminergic genes.

## Introduction

Attention can be directed to spatial locations either voluntarily (endogenous cueing) or reflexively (exogenous cueing). There is currently a sense that the reflexive aspect of attentional orienting is related to cholinergic neurotransmitter genes but only weakly or not at all to variations in dopaminergic genes [1], [2]. However, many of the previous studies showing associations with cholinergic genes used endogenous cues to direct attention spatially, so it is at least possible that the use of exogenous cueing to produce more reflexive orienting might result in additional associations with dopaminergic genes. Additionally, the costs and benefits of using invalid and valid spatial cues, respectively, were not analyzed separately in these previous experiments. Examining costs (increases in response time, RT, following an invalid pre-cue) and benefits (decreases in RT following a valid pre-cue) separately could be important if dopaminergic genes (implicated in disorders of attention such as attention deficit disorder; *ADHD*) influenced the different processes of attentional orienting independently (e.g., disengaging, moving, re-engaging attention; [3]).

Visual orienting is impaired in many heritable disorders including Alzheimer's disease, autism, and schizophrenia [4]–[7]. Genes control the synthesis, transportation, conversion, and metabolism of neurotransmitters and therefore offer plausible biological pathways for understanding these disorders. Both dopamine [8] and acetylcholine [9] have been implicated in attention processes generally. In this paper we examine possible associations between several genetic markers (“markers”) and visual orienting using a modification of Posner and Cohen's exogenous cued orienting task [10]. In addition, we tested the hypothesis that genetic associations with visual orienting might only be detected when attentional costs and benefits are analyzed separately rather than combined into a single validity score (e.g., mean benefit minus mean cost).

Although visual orienting is an important component of many heritable disorders, studies have not always found genetic associations with orienting. For example, Fan et al., [11] failed to find significant heritability for visual orienting in a twin study. However, their task did not use invalid cues to calculate the cost component of exogenous orienting. We feel that it is important to test genetic associations with this aspect of orienting. Posner, Walker, Friedrich & Rafal [3] described three steps in orienting: engage, disengage, and move. Looking at costs and benefits separately allows for examination of possibly separate genetic influences on all three aspects of orienting as proposed by Posner and colleagues. The response to a target following a valid pre-cue only requires the engage function since attention is already at that location. In particular, lower availability of dopamine (as determined by genotype) could lead to significantly larger costs because the availability of dopamine could impact the rapid reorienting of attention.

In addition to the Fan et al., study [11], a more recent review notes that associations between dopaminergic genes and orienting have generally been lacking [1]. In contrast, associations between visual orienting and cholinergic genes have been found in normal populations [2], [12]. Like the differences between tasks described above, the tasks in these studies were also somewhat different from the current task in that we used exogenous cues, while the tasks of Espeseth et al., [12], Fan et al., [11], and Parasuraman et al., [2] used endogenous cues. Here, we re-examined possible relations between exogenously-cued (reflexive) visual orienting and several genetic markers (one cholinergic, one noradrenergic, and three dopaminergic). The noradrenergic gene, dopamine beta hydroxylase (*DBH*), is related to dopamine in that it converts dopamine to norepinephrine (noradrenaline). We added several conditions (see below) to the standard cued-orienting procedure in an attempt to employ more sensitive behavioral measures of orienting in the genetic association analysis.

We reasoned that if *costs* and *benefits* were even partially determined by distinct neural mechanisms, then this would argue for separate analysis when searching for genetic associations. Treating costs and benefits separately would be consistent with Posner's formulation of orienting as a three-step process in which disengaging attention is necessary for invalid but not for valid cues.

### Genetics and Visual Attention

There are several ways to select genes for study. One method, genome-wide association, scans the entire genome for association with a phenotype. This is potentially problematic because it increases the risk of Type I errors unless very large sample sizes are used. One way to avoid this problem is to select candidate DNA markers based on biological pathways such as those that control key neurotransmitters [13]. In selecting genetic markers, we searched the literature and considered genes that controlled neurotransmitter availability and/or were associated with diseases having an attentional component.

We narrowed this list to the current selection of markers based on such factors as the availability of a precise marker location (such as a ‘reference SNP;’ single nucleotide polymorphism), the allele in question having a known impact on biological function, a moderate to large effect size, and/or the existence of a relationship with a disease having a purported reflexive orienting component.

In planning our analysis, we tried to avoid common problems in genetic association studies. One challenge is that variation on a single marker rarely accounts for more than 5%–10% of the variance in a complex phenotype which may be influenced by many genetic and environmental factors. This will make the association statistically difficult to detect without large sample sizes [14] or very precise measures. One way to increase precision is to use endophenotypes (specific phenotypic measures) rather than more global constructs [15]. We used outcomes derived to measure specific aspects of visual orienting. In addition to standard orienting, we also examined alerting, another aspect of attention, but found no significant associations. In the selection of genetic markers, we also examined prior research for biological and functional effects (see Table 1). Both the use of endophenotypes and careful selection of genetic markers increased the likelihood of finding valid associations.

**Table 1:** Biological and Functional Effects of Genetic Markers in this Study.

Genetic Marker	Risk Allele	Biological Effect	Functional Effect
*COMT* rs4680	G	G at rs4680 produces valine which is more active in catabolizing dopamine and so less dopamine is available [18].	Reduced cognitive function [18].
*DAT1* intron 8 VNTR	6R	6R leads to more dopamine transporter and therefore less dopamine in the synapse [20], and this terminates the dopaminergic signal transmission [21].	Greater cuing costs for targets in the left hemifield [19].
*DRD4* rs747302	C	C leads to fewer dopamine receptors via reduced transcription [27].	There is an association between rs747302 and ADHD [27].
*APOE*	e4	e4 reduces acetylcholine receptor number [25] and possibly diminished synthesis of acetylcholine via impaired regulation of phospholipids and/or fatty acid transport [26].	Middle age, nondemented carriers of e4 showed deficits in spatially cued visual tasks [23].
*DBH* rs1108580	A	DβH converts dopamine to norepinephrine and the A allele is associated with lower levels of plasma DβH [28] and therefore lower norepinephrine to dopamine ratios [53].	Lower levels of plasma DBH activity have been associated with attention deficit [28].

*Note.* Bellgrove et al. [19] refer to 3R but according to Rommelse et al. [22] 3R is now called 6R.

Another problem (stratification) occurs when there are systematic differences in a phenotype that have nothing to do with the marker under study, yet the association appears statistically significant. These spurious associations can arise when ethnic groups are combined in the same study, differ on a phenotype and simultaneously differ for unrelated reasons on the target genotype frequencies. To address this problem we controlled for ethnicity in the statistical analyses ([16]; see also the Discussion section). Additionally, unlike genome-wide scans in which no *a priori* markers have been selected, we specifically chose the markers in the present research for their known biological or functional effects thus reducing the risk of spurious associations.

Here we report the results for five genetic markers studied for association with visual orienting. The markers were: *COMT* Val158Met (NM_000754.3; a SNP, rs4680; [17], [18]), *DAT1* (*SLC6A3*; NM_001044.4; a 30 bp VNTR on intron 8; [19]–[22]), and APOE (NM_000041.2; a two SNP composite, rs429358 and rs7412; [23]–[26]), *DRD4* (NM_000797.3; a SNP, rs747302; [27]) and *DBH* (NM_000787.3; a SNP, rs1108580; [28], [29]). Although the SNP in *DBH* showed no significant associations with any of the attention measures, we included it in this report because it shows that not all markers related to dopamine were associated with our attentional measures. (See Table 1 for more details on the biological and functional rationale for selecting these genetic markers).

## Results

We only included data in the following analyses from subjects who made errors on 5% or fewer of the 200 experimental trials. In addition, only a subject's correct trials were included. An average of 1.44% of trials were excluded due to error.

As stated previously, the derived measures used in the analyses are difference scores. All derived measures were significantly different from zero with the exception of *benefit dim* (*p = *.98) and *dual asymmetric cost bright* (*p* = .42), and all of the derived measures were in the expected direction (that is, benefits were positive and costs were negative; with an average *SEM* = 2.30 msec). Thus, the basic paradigm produced significant cueing effects including the standard alerting effects as well as costs and benefits from invalid and valid cues, respectively. We also found, as Kean and Lambert [30] did, that when bright and dim cues appeared simultaneously in contralateral spatial locations, subjects responded more quickly (by 23.93 msec, *SEM* = 1.92 msec) to a target when it appeared near the location of a preceding brighter cue than when it appeared near the location of the dimmer cue. Thus, our use of dual asymmetric luminance cues produced the expected RT difference.

One of the key questions in this study was whether RT costs and benefits were correlated. There was a statistically significant correlation between *benefit bright* and *cost bright* (*r* = .28, *p* = .001) and, likewise, between *benefit dim* and *cost dim* (*r* = .23, *p* = .01). Because benefits are coded positively and costs are coded negatively, these correlations indicate that larger costs were associated with smaller benefits. The magnitudes of these correlations of costs and benefits indicate that only 7.78% of the variance in benefits based on *bright* cues and 5.51% of the variance in benefits based on *dim* cues is explained by knowing the corresponding cost measure. We took this as support for treating costs and benefits separately in the subsequent genetic association analyses rather than combining them into a single validity score.

### Genetic Associations

Because we had 10 outcome measures, we initially conducted a MANCOVA with age and ethnicity as covariates and all five genes as predictors. We used Roy's largest root to determine statistical significance because of the high inter-correlations between the dependent variables and because the first canonical variate for each gene explained a high percentage of the variance in the outcome measures (greater than 72%). Four of the five genes were statistically significant after controlling for age and ethnicity: *DRD4* (*F*[10, 86] = 1.89, *p* = 0.06); *DAT1* (*F*[10, 86] = 2.64, *p* = 0.007); *COMT* (*F*[10, 86] = 2.27, *p* = 0.02); *DBH* (*F*[10, 86] = 0.77, *p* = 0.65); and *APOE* (*F*[10, 86] = 1.96, *p* = 0.047). The remaining analyses were conducted as described in the methods section. That is, an initial ANOVA was used with ethnicity as an independent variable to control statistically for possible population stratification artifacts. To test for genetic associations, genotype was then added as another independent variable, and the incremental *R* ^2^ was obtained.

**Figure 1:**
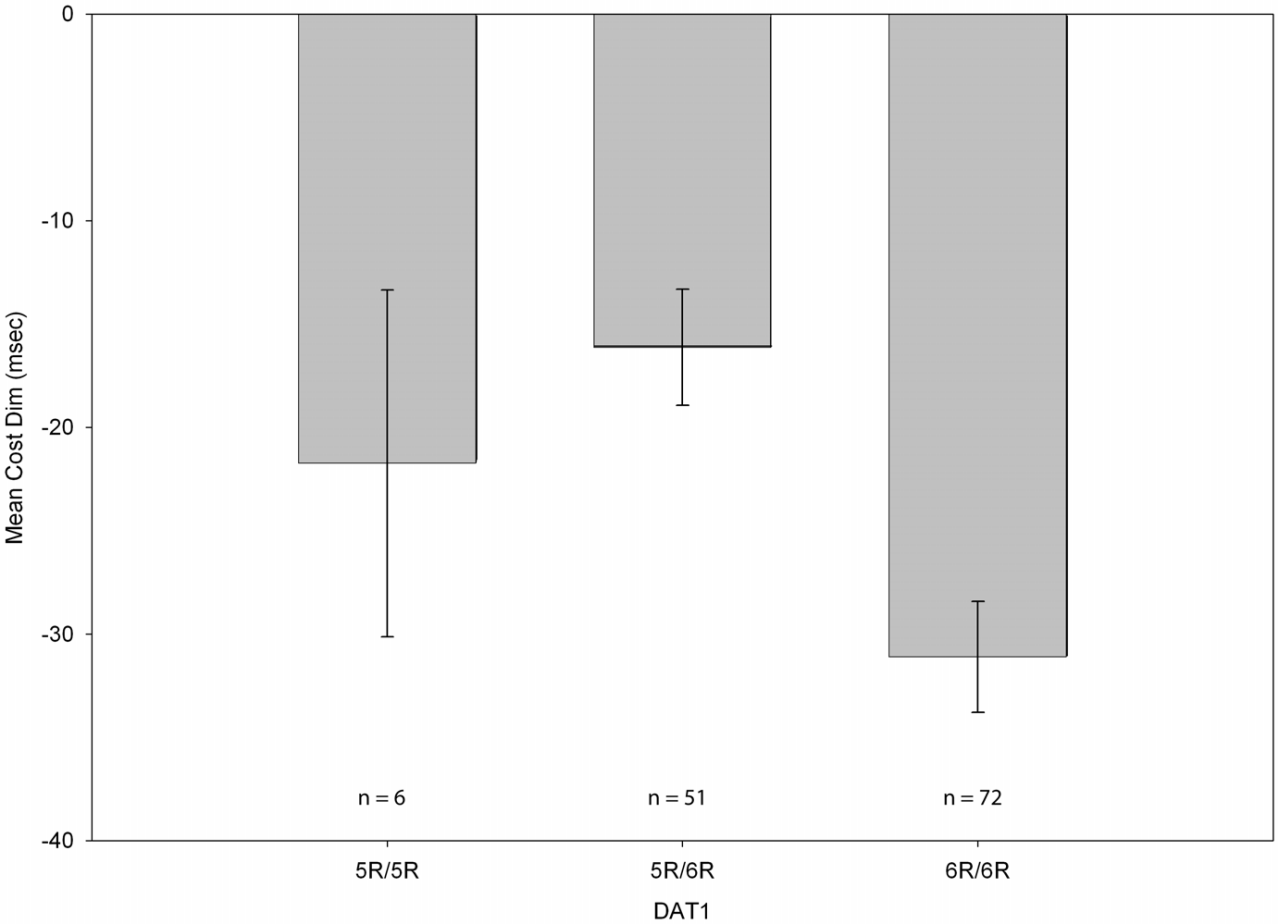
Mean *cost dim* difference scores by *DAT1* genotype. Note that the 5R/5R genotype group had only 6 subjects and the mean may, therefore, not be reliable. This graph represents the mean after adjustment for ethnicity. Error bars are +/−1 SEM.

*DAT1* (30 bp VNTR on intron 8) was associated significantly with *cost dim* (*R^2^* change = 8%, *F* [1, 124] = 11.97, *p*<.001) but not with the *cost bright* (*p* = .46) or *benefit dim* measures (*p*>.99). *Cost bright* and *benefit dim p*-values are provided for comparison purposes as the lack of significance for these measures suggests the importance of including separate analyses of both costs and benefits and of cue luminance levels. The 6-repeat/6-repeat (6R/6R) genotype at this location was associated with larger *costs* following *invalid dim* cues (Figure 1).

**Figure 2:**
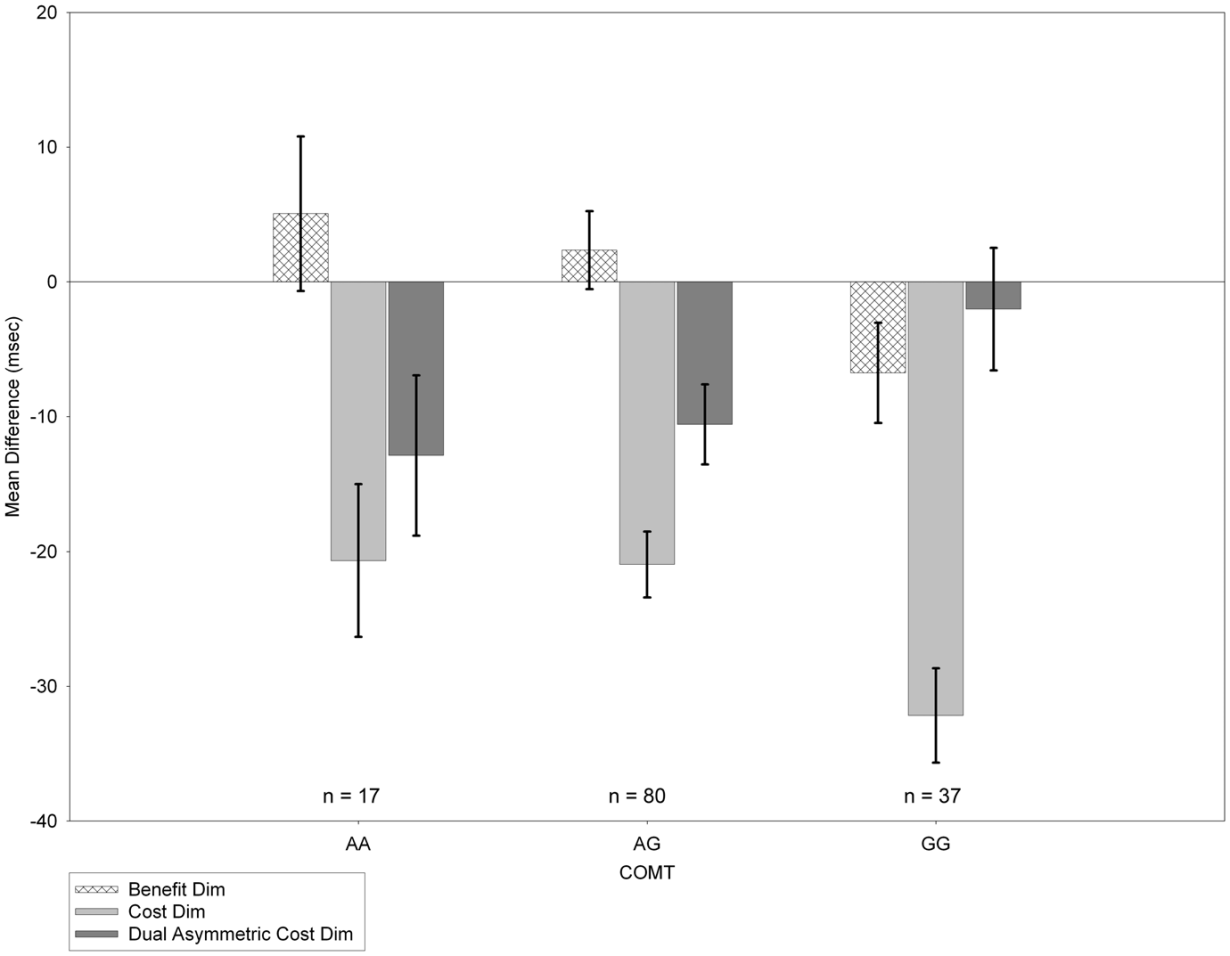
Mean *cost dim benefit dim*, and *dual asymmetric cost dim* difference scores by COMT genotype. This graph represents means after each measure was adjusted for ethnicity. Error bars are +/−1 SEM.

*COMT* (rs4680) was also associated significantly with the *cost dim* measure (*R^2^* change = 4%, *F* [1, 129] = 5.40, *p* = .02) meaning the GG genotype (Val/Val) at this location was associated with larger *costs* following *invalid dim* cues. *Benefit dim* likewise showed significant association (*R^2^* change = 3%, *F* [1, 129] = 3.97, *p* = .048; Figure 2), but *cost bright* did not (*p* = .60). *Dual asymmetric cost dim* did not reach conventional significance levels (*R^2^* change = 2%, *F* [1, 129] = 2.93, *p* = .09) but is reported here as a guide to future research.

**Figure 3:**
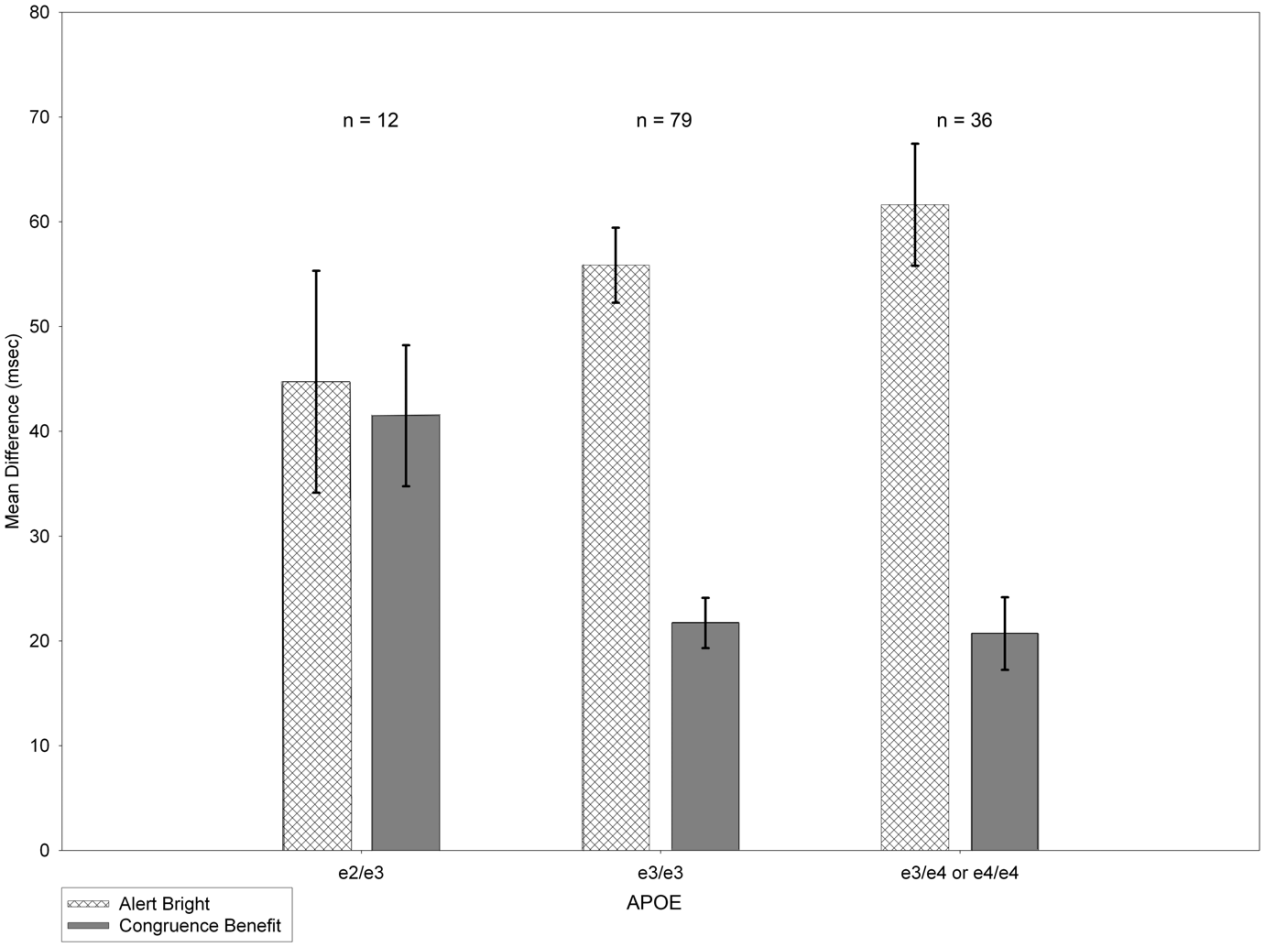
Mean *congruence benefit* and *alert bright* difference scores by *APOE* genotype. With two SNPs contributing to APOE status, there are six possible groups. We organized these into groupings as did others [23], however we modified the groups by eliminating subjects who had one risk allele (e4) and one protective factor (e2) (*n* = 2) since a hypothesis for the outcome in such individuals was unclear [24]. The sample size for the e2/e3 genotype is relatively small, but represents a naturally occurring group with a putative protective factor. The e2 allele is relatively rare. For example, in a sample of 5000 alleles from 2500 subjects only 7.65% of the alleles were e2 [24]. This graph represents means after adjustment for ethnicity. Error bars are +/−1 SEM.

Those with the e2/e3 genotype of *APOE* were faster at responding following bright than dim cues, as measured by *congruence benefit*, in contrast to those with e3/e3 or the combined e3/e4 and e4/e4 genotypes (Figure 3). The differences are significant, *R* ^2^ change = 5%, *F*(1, 120) = 6.88, *p* = .01. *Congruence benefit* measures the extent to which subjects respond faster to a target when it appears near the brighter of two simultaneous cues placed symmetrically with respect to fixation [30]. It is not possible to detect this effect with a single cue paradigm. *Alert bright* did not reach conventional significance levels, *R* ^2^ change = 2%, *F*(1, 120) = 2.61, *p* = .11.

**Figure 4:**
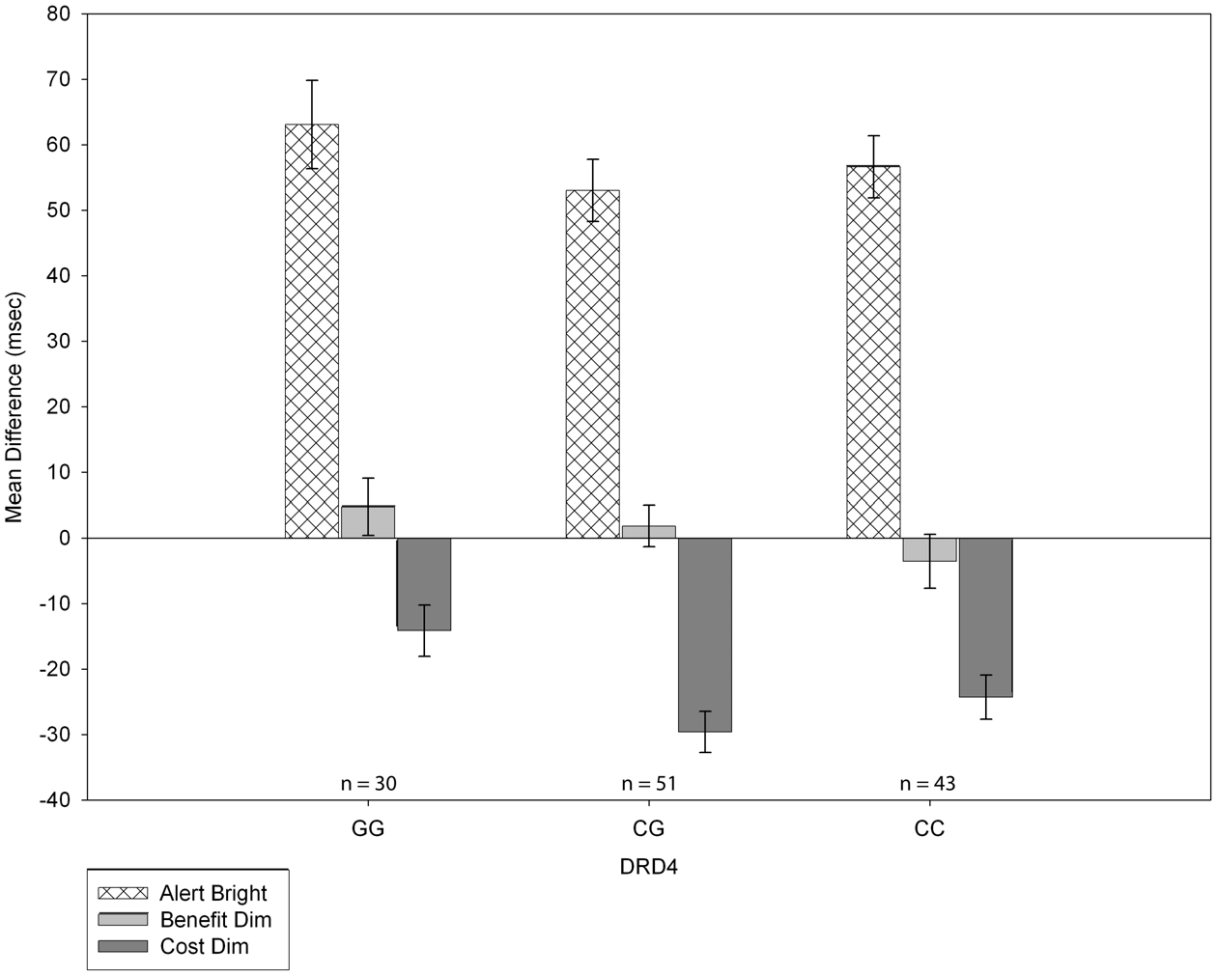
Mean *cost dim*, *benefit dim*, and *alert bright* difference scores by *DRD4* genotype. This graph represents means after adjustment for ethnicity. Error bars are +/−1 SEM.

*Alert bright* showed a weak association with genotype on *DRD4*, *R^2^* change = 3%, *F*[1, 119] = 3.37, *p* = .07. Neither *benefit dim* (*R^2^* change = 2%, *F*[1, 119] = 2.11, *p* = .15) nor *cost dim* (*R^2^* change = 2%, *F*[1, 119] = 2.56, *p* = .11; Figure 4) reached conventional significance levels by genotype on *DRD4*. However, those with the GG genotype tended toward less of a cost to a *dim*, *invalid* cue than those with CG or CC genotypes. They also showed greater benefits to a *dim*, *valid* cue than those with the CC genotype. Table 2 summarizes these effects.

**Table 2:** Effect Sizes (R^2^ change) for Significant Associations after Controlling for Ethnicity.

Genetic Marker	N	Measure	R^2^ change	F(1, error df complete)	p-value
DAT1 30 bp VNTR on intron 8	131	cost dim	0.09	12.71 (1, 126)	<.0001
*COMT* rs4680	136	cost dim	0.04	5.57 (1, 131)	0.02
*COMT* rs4680	136	benefit dim	0.03	4.17 (1, 131)	0.04
*APOE* rs429358 & rs7412*	127	congruence benefit	0.05	6.95 (1, 122)	0.01
*DRD4* rs747302	126	cost dim	0.02	2.44 (1, 121)	0.12
*DRD4* rs747302	126	benefit dim	0.02	2.53 (1, 121)	0.11

*Note*. APOE status is determined jointly by two SNPs.

Finally, no significant results were found for *DBH*.

## Discussion

The results lead to three conclusions. First, the luminance of a cue significantly influenced the likelihood of observing an association between a genetic marker and specific attentional measures. Second, attentional costs and benefits should be analyzed separately when examining genetic associations. The alternative, combining them into a single validity score, runs the risk of missing differential genetic associations on these alternate measures of visual orienting. Third, in contrast to several previous studies, we find that dopaminergic markers on *DAT1* and *COMT* showed significant associations with specific orienting measures. *APOE* also showed a significant association with a specific orienting measure. We will elaborate briefly on these conclusions.

In the case of our conclusion regarding luminance, we note that for *DAT1* and *COMT cost dim* reached significance, but *cost bright* did not. This suggests that the luminance of the cues matters in assessing gene-attention associations. Consider several explanations for this result. First, it should be noted that RT variance was approximately equal at the two cue luminance levels, thus ruling out reduced RT variance as an explanation for the lack of an association with the brighter cues. It should also be kept in mind that the luminance of the target was always the same in all conditions; it was only the luminance of the cues that differed. Second, consider the possibility that dim cues might require more attentional effort thereby recruiting executive aspects of attention. This does not seem likely because subjects were dark adapted, and the dim cues were clearly visible, although obviously dimmer than the bright cues (luminance ratio 5.85∶1). We also note that subjects were explicitly instructed to ignore the cues because they did not predict the location of the target across the set of trials. Under these circumstances, it seems unlikely that differences in cue luminance would have exerted their effects by requiring more or less attentional effort.

Third, consider the possibility that the validity manipulation might have induced conflict processing, also recruiting more executive aspects of attention. Specifically, including invalid trials means that on some trials there is a conflict between the location of a cue and the location of a target, while on other trials there is not. Nevertheless, even though cues do not predict the location of the target (that is, cues are 50% valid and 50% invalid) the task clearly taps reflexive processes since it still produces the expected costs and benefits. Conflict processing is unlikely to be involved because exogenously cued orienting is generally regarded as being reflexive [31]–[33].

There is some empirical evidence that the level of dopamine interacts with cue intensity to determine reactions times. On the sensory side, dopamine release is influenced by stimulus intensity at early stages in the visual system [34], [35]. On the attentional side, Rihet, Possamaï, Micallef-Roll, Blin, & Hasbroucq [36], in particular, showed that when levodopa versus a placebo was administered to subjects, RT decreased more (i.e., the difference was greater) with weak intensity cues than with strong intensity cues. In other words, weak intensity cues were better at revealing the impact of a dopamine agonist on RT. This is very similar to our results in the sense that when we found associations with dopamine related markers it was only with our low intensity (dim) cues.

When we examined the costs for bright versus dim cues with the *COMT* gene, we found an interesting pattern of results that could explain why the intensity of a cue matters when testing for genetic associations. The intensity of an invalid cue did not affect the time that it took subjects with the GG (less dopamine) genotype to respond to the appearance of the target on the side contralateral to the cue; the average costs relative to their respective neutral baseline conditions were approximately 30–35 msec regardless of the intensity of the cue. Interestingly, subjects with the AA (more dopamine) genotype also showed an average cost in this same range (32 msec) when a bright, invalid cue was used. In contrast, subjects with the AA genotype showed an average cost (18 msec) that was approximately half as large when a dim, invalid cue was used. It should also be noted that within each of these genotypes the latency to respond to the target did not depend on the intensity of the cues when neutral cues were used (that is, two, equal luminance cues presented simultaneously). This shows that those with the AA genotype on *COMT* are able to disengage and shift attention more quickly than those with the GG genotype but only when *weaker* invalid cues are used. For both genotypes, the costs were approximately the same when a *stronger* invalid cue was used, so the result cannot simply be attributed to a superior overall ability of those with the AA genotype to disengage attention from an invalid cue more quickly. This suggests that the reason that we observed genetic associations with attentional costs when dim cues were used but not when bright cues were used is that the level of available dopamine only has an effect on how long *weak* cues distract or hold attention. Apparently, those with the GG genotype on *COMT* are just as distracted by these weak, irrelevant cues as they are by the stronger ones while those with the AA genotype are less distracted by weaker cues.

To understand these results, assume a simple model like that considered by Luce and Green [37] of how subjects make a forced-choice, speeded decision that results in a right vs. left response to the target. To make this decision about the side on which the target appeared, subjects are assumed to sample incoming neural information from the regions around the two locations in which the target can appear. Once sufficient information has been accumulated to allow the subject to discriminate these two samples, the decision is reached, and the subject responds right vs. left. A critical aspect of the model proposed by Luce and Green [37] is that the subject must start this process of sampling neural information from the two likely target locations at some point in time relative to the onset of the target. If one assumes that the appearance of the cue automatically biases the subject to start sampling from the possible target location ipsilateral to the cue earlier than from the contralateral location, then one would expect faster RTs when the target subsequently appeared on the side ipsilateral to the cue and slower RTs when the target appeared on the side contralateral to the target, thus producing the typical pattern of costs and benefits. Notice, however, that because the cues are completely uninformative in a design like the one that we used, these automatic biasing effects would only be helpful (in terms of the latency to respond to a target) if the benefits were on average greater than the costs. In our data, the costs were typically larger than the benefits for a given cue intensity, so being able to ignore these cues, or being able to disengage from them more quickly to start sampling from both target locations simultaneously would be a better strategy on average. Our results appear to show that subjects with the AA genotype on the *COMT* come closer to being able to ignore or to disengage more quickly from weak, irrelevant (distracting) cues than do subjects with the GG genotype. Apparently, this must be more difficult to do when stronger peripheral cues are used because both genotypes showed approximately the same attentional costs under those conditions.

This result is similar, but not identical, to an effect discussed by Nissen [38] showing that *target* intensity had an effect on attentional costs but not on attentional benefits such that costs were greater with lower intensity targets than with higher intensity targets. Nissen concluded that when intensity had an effect in these cueing paradigms, it tended to be on the attentional mechanisms rather than on the sensory pathways. While our effect involved the intensity of the *cues* rather than of the *targets*, the two sets of results are similar in that when intensity exerted an effect, in both cases the locus of the effect appeared to be at the level of attention rather than at the level of the sensory pathway, and the effect was observed with attentional costs but not with attentional benefits.

In addition to suggesting the importance of luminance, the fact that, for *DAT1*, *cost dim* is significant but *benefit dim* is not argues for separating costs and benefits. Had we used only a validity score, we would have missed the fact that benefits and costs were differentially associated with individual differences in various intake measures (e.g. age, ethnicity, and tobacco use; see Table 3) as well as with genotypic variation on *DAT1*. That costs and benefits should be separated is also suggested by their low correlations (mentioned above; see Table 4). This is despite the fact that each was calculated as a difference from the same baseline measure (RT to dual neutral cues). When the cost of attending to an invalid cue is not separated from the benefit of attending to a valid cue, then the single validity measure could be less sensitive in detecting subtle individual differences. Using a combined validity measure provides inherently less information.

**Table 3:** Correlations between Intake Variables and Outcome Measures.

Variable	AB	AD	BB	BD	CB	CD	CongB	DACB	DACD	DBTN	VEbright	VEdim
Location	−0.07	0.00	−0.16*	−0.12	0.04	0.06	0.37***	0.32***	−0.15*	−0.11	−0.17*	−0.14*
Age	−0.05	0.00	−0.19*	−0.16*	0.02	0.05	0.22*	0.25**	−0.03	−0.07	−0.17*	−0.17*
Gender	0.04	−0.01	0.02	0.10	0.15*	0.13	−0.13	0.03	0.08	0.00	−0.12	−0.01
Asian	−0.03	−0.12	0.08	0.15*	−0.12	0.00	−0.25**	−0.23**	0.07	0.19*	0.17*	0.13
Black	−0.12	−0.15*	−0.02	0.02	−0.05	−0.17*	0.05	0.04	−0.03	0.06	0.03	0.14*
Hispanic	−0.01	−0.01	−0.02	−0.01	−0.20*	−0.14	−0.06	−0.02	0.03	0.21*	0.15*	0.09
sleepiness	0.09	0.15*	0.03	−0.14	0.02	−0.10	−0.02	−0.02	0.08	−0.02	0.00	−0.04
tobacco	−0.02	0.02	−0.18*	−0.06	0.02	−0.06	−0.09	0.15*	0.09	−0.10	−0.16*	0.00

Note. AB = Alert Bright; AD = Alert Dim; BB = Benefit Bright; BD = Benefit Dim; CB = Cost Bright; CD = Cost dim; CongB = Congruence Benefit; DACB = Dual Asymmetric Cost Bright; DACD = Dual Asymmetric Cost dim; DBTN = Dim Better Than Nothing; VEdim = Validity Effect Dim; and VEbright = Validity Effect Bright. Asian, Black and Hispanic ethnicities are each compared to the White ethnicity.

* = p<.10;

** = p<.01; and.

*** = p<.001.

**Table 4:** Inter-Correlations between Outcome Measures.

Measure	AB	AD	BB	BD	CB	CD	CongB	DACB	DACD	DBTN	VEbright	VEdim
AB	1	0.77***	−0.28*	−0.03	−0.27**	0.09	−0.16*	−0.16*	0.11	0.04	0.01	−0.09
AD		1	−0.06	−0.40***	0.07	−0.16*	−0.21*	−0.13	0.21*	−0.03	−0.11	−0.21*
BB			1	0.18*	0.28**	−0.12	0.09	−0.69***	−0.25**	−0.12	0.56***	0.24**
BD				1	−0.19*	0.23**	0.11	−0.26**	−0.65***	0.00	0.31***	0.66***
CB					1	0.19*	−0.12	0.05	0.16*	−0.56***	−0.64***	−0.31***
CD						1	−0.03	0.07	0.04	−0.29**	−0.26**	−0.59***
CongB							1	0.29**	−0.55***	−0.28**	0.18*	0.12
DACB								1	0.24**	0.05	−0.60***	−0.27**
DACD									1	0.19*	−0.34***	−0.59**
DBTN										1	0.39***	0.23**
VEbright											1	0.46***
VEdim												1

*Note*. AB = Alert Bright; AD = Alert Dim; BB = Benefit Bright; BD = Benefit Dim; CB = Cost Bright; CD = *Cost dim*; CongB = Congruence Benefit; DACB = Dual Asymmetric Cost Bright; DACD = Dual Asymmetric *Cost dim*; DBTN = Dim Better Than Nothing; VEdim = Validity Effect Dim; and VEbright = Validity Effect Bright. *p*-values are below each correlation. *N* = 143. Costs are coded negatively.

* = *p*<.10;

** = *p*<.01; and.

*** = *p*<.001.

We also note that the percentage of variance on *cost dim* that was statistically explained by variance on *benefit dim*, 5%–7%, is approximately the same magnitude as the percentage of variance explained by the genetic markers that were statistically significant. In other words, knowing a subject's genotype on a particular marker predicts their cost measure approximately as accurately as knowing that subject's benefit measure. We find it remarkable that genetic information does approximately as good a job at predicting a subject's behavioral measure as does another contemporaneously collected behavioral measure on that same subject.

Our third conclusion is that *DAT1*, *COMT*, and *APOE* each showed significant associations with specific attentional measures derived from this visual orienting paradigm (neither *DBH* nor *DRD4* reached conventional significance levels). The *cost dim* measure was particularly useful in this regard. These significant genotype-phenotype associations stand in contrast to previous reports showing associations for more executive aspects of attention but not for orienting [11], [39] or those that suggest that cholinergic genes are associated with orienting but dopaminergic genes are not [1], [2]. One caution to this conclusion is that some of the dopaminergic markers that we studied could affect cholinergic processes and thus exert their effects indirectly [40]. Further research is needed to clarify this issue.

There is always the possibility of Type I errors in genetic association studies. However, we think it unlikely that our results are entirely spurious for several reasons. First, there is both a plausible biologic pathway between dopamine availability and attention and a dose-response relationship in our data (tested with our linear model). These are often considered the minimum requirements in searching for legitimate associations [41]–[43]. Second, with two of the four markers for which we found significant associations, the same behavioral measure, *cost dim*, proved significant. Had our results been primarily Type I error, the statistically significant effects most likely would have been randomly distributed across the behavioral measures. Third, because the markers that we selected for analysis were chosen based on prior association with attention, our findings (including the direction of observed effects following putative risk) can be considered a constructive replication. Replication is unlikely if the results are spurious.

Population stratification is a potential problem precisely because it may lead to an increased type I error rate, but it deserves additional consideration. First, absent gene-gene and/or gene-environment interactions, knowledge of a biological pathway can be helpful in reducing the risk of threats to validity from population stratification. For other reasons, however, our results are unlikely to be due to population stratification. Recall that, for population stratification to exist, substantial phenotypic differences must be present between ethnic groups [44]. However, there is not significant inter-ethnic variation in the *cost dim* measure; therefore the current findings are unlikely to be the result of population stratification. Several authors have determined that even in cases where conditions exist that make population stratification a possibility, potential bias remains small [45], [46]. Nevertheless, we statistically controlled for ethnicity which has been shown to be effective [16].

In conclusion, *COMT*, *DAT1*, and *APOE* all showed associations with specific measures designed to study visual orienting. These results stand in contrast to those of others [11] who concluded that visual orienting shows little heritability. It is possible that the use of weaker cues produced a more sensitive measure of orienting, thereby allowing us to detect these subtle genotype-phenotype associations.

## Materials and Methods

### Ethics Statement

The Institutional Review Board of Rice University approved this study. Written consent was obtained from each participant prior to the experiments, and the experiments adhered to the principles found in the Declaration of Helsinki.

### Participants

We tested normal subjects (*N* = 161) between the ages of 18 and 61 years (69 males). Most of the participants (*n* = 108) were Rice University students. A community sample was also obtained (*n* = 53) to increase the age range of the total sample. Prior to completing the visual orienting task, subjects signed a consent form and completed an intake questionnaire that included questions on basic demographics, attentional disorders in self and biological relatives, tobacco use, and the Epworth Sleepiness Scale [47]. Based on the distribution of error rates, a subject's data were excluded from analysis if they had greater than 5% errors (including catch trial errors). It makes sense to exclude those subjects who have high error rates because it indicates that they might not be motivated or might not have understood the task. The median error rate for those subjects whose data were excluded was 9% (range = 6%–31%). A subject's data were also excluded if they had a history of a serious neurological disorder. The data from two subjects (both from the community sample) were discarded because of a history of a neurological disorder. Subjects were not excluded if they had a current diagnosis of ADHD. Eight subjects reported this diagnosis, four of whom were on medication. The pattern of results was substantially the same when the analyses were run without these subjects and so we included them. Ninety-one of 108 participants in the Rice University sample had useable data (45.05% male) as did 46 of the 53 community participants (43.48% male). Overall, 85.09% of the subjects had useable data and the final sample consisted of 137 subjects. The mean age for the university sample was 20.52 years (range 18 to 44 years), and for the community sample it was 35.11 years (range 18 to 61 years). Age did not make a difference in the results (see Results section).

### Behavioral Task Procedures and Stimuli

Participants were dark adapted before beginning the behavioral task and completed 20 practice trials before beginning data collection trials. They viewed a 1024×768 pixel CRT monitor with a background luminance of 0.08 cd/m^2^. A fixation cross, centered on the monitor, was always visible. Participants were instructed to fixate the central cross and to maintain fixation throughout data collection.

We used both single and dual spatial pre-cues. Dual (bilateral) cue conditions were similar to those used by Kean and Lambert [30]. Kean and Lambert showed that observers were faster to saccade to a target that appeared near the brighter of two simultaneously presented, but spatially separated pre-cues compared to when the target appeared near the dimmer of the two cues. We added these dual, unequal cue luminance trials to the standard single cue conditions (both valid and invalid) because we hypothesized that they might be more sensitive to individual differences in the ability to split attention or allocate attention to differentially salient locations. To provide baseline results against which to compare these dual, unequal cue luminance trials, we also included single cue trials using either the brighter or the dimmer of the cues. We fixed the cue-target stimulus onset asynchrony (SOA) at 150 msec based on pilot testing of the dual asymmetric cues. This SOA is also in line with prior literature on exogenously cued orienting [32], [48]–[50]. Otherwise, the luminances, sizes, timing, and location of our stimuli were identical to those reported in Kean and Lambert [30].

**Figure 5:**
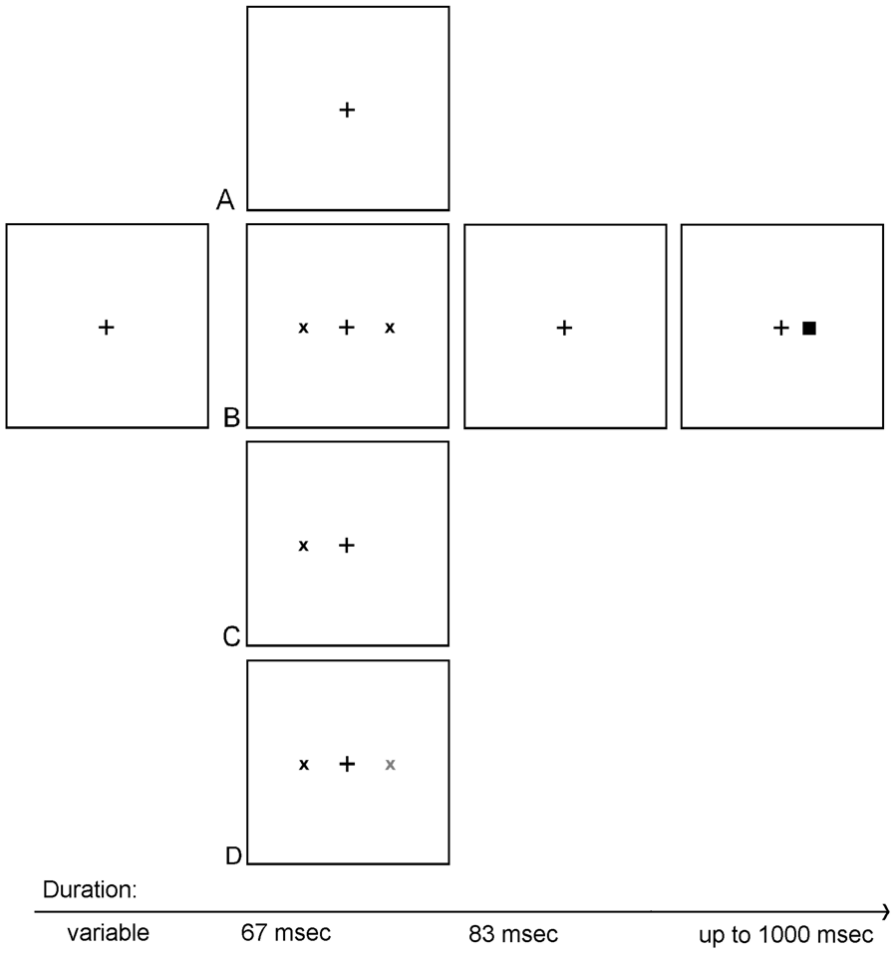
Illustration of stimuli: The fixation cross was visible throughout a trial. A pre-cue condition appeared for 67 msec. Conditions a) no cue and b) dual cues of a single luminance were used as baseline conditions for the calculations of derived measures as described in Table 1. Otherwise, either one or two cues of different luminances appeared. A single target as in condition c) could appear on either the left or right side with 50% probability and could be either bright or dim (50% probability each). The target that followed could appear near where the pre-cue had appeared or contralaterally. If two cues appeared as in condition d) then the bright cue would appear on the left side for 50% of these trials. After the cue condition disappeared, a single target followed and could appear near the dim or the bright cue.

One or two cues were presented for 67 msec. The cues could be valid (i.e., appear where the target would subsequently appear) or invalid (appear contralateral to where the target would subsequently appear). There was an 83 msec gap after the offset of the cues and prior to the onset of the target. The target remained on display for 1000 msec or until the participant made a key press (see Figure 5). Participants were asked to respond as quickly as possible while maintaining accuracy by making a key press to indicate a target either to the left (pressing ‘a’) or to the right (pressing ‘l’) of fixation. After the participant responded, there was a variable delay (1.3 to 1.8 sec), and the next trial began. No feedback was provided.

Single cue trials were intermixed with catch, dual neutral, and dual asymmetric cue trials. The side on which the cue appeared (for single trials) or on which the brighter cue appeared (for dual asymmetric cue trials) was random, and each appeared on the right side of the display for 50% of the trials. The target also appeared randomly on half the trials on the right side of the display independently of where the cues appeared. Participants were told 1) that the cues did not predict the target's location and 2) to ignore the cues as much as possible. Participants completed all trials within one session with pauses as necessary. RT was measured from the onset of the target.

Dual asymmetric cues comprised two cues of unequal luminance presented on either side of fixation. The brighter and dimmer cue luminances were 11.7 and 2.0 cd/m^2^, respectively. The target (a square) always had a luminance of 15.5 cd/m^2^. The centermost edge of the target appeared 5.5 deg to either side of the fixation cross. The cues were shaped like the letter X, measured 0.8 (width)×1.0 (height) deg, and appeared 7.3 deg (innermost edges) to the left and right of the display's center.

#### Primary measures

There were nine primary RT measures. These measures were computed as the average RT (msec) for correct responses to different cue-target conditions. *Single dim valid* indicates a single, dim luminance cue followed by a target near where the cue was presented. The term ‘valid’ indicates that the target appeared near that location shortly after the offset of the cue. Conversely, the configuration termed *single dim invalid* indicates a dim cue followed by a target on the side contralateral to where the cue was presented. There were corresponding valid and invalid configurations for the *single bright* cues. We also included *neutral bright* and *neutral dim* cues. On these trials, identical bright or dim cues were presented simultaneously on both sides of the fixation cross. These spatially neutral cues were used to calculate alerting effects. When the *dual asymmetric* cues were presented, the target could appear either near the brighter cue (*dual asymmetric bright*) or near the dimmer cue (*dual asymmetric dim*). Finally, targets could appear uncued without being preceded by any cues. Catch trials were also presented, and subjects were instructed to withhold responding since no target appeared.

Incorrect responses, responses made before 200 msec or after 1000 msec from the onset of the target and responses to catch and practice trials were not included in the analysis. Each of the 10 (nine target-present plus one target-absent) conditions was presented 20 times, yielding 200 trials. For analysis, the subject's mean RT to correct trials was determined separately for each of the nine target-present conditions.

#### Derived measures

We derived 10 measures by computing within-subject differences between selected pairs of the primary measures. Three of these are standard measures in a Posner-type cueing paradigm: alerting, costs, and benefits. Costs, however, were coded negatively because we felt this better reflects the different direction of the effect on RT. The use of two different cue luminances yielded six of these three standard, derived measures. We derived four additional measures by using trials in which the dual, asymmetric luminance cues appeared. Table 5 shows the differences between primary measures that produced these 10 derived measures. These measures served as the endophenotypes in the association analyses.

**Table 5:** The Calculation of Derived Measures.

Derived Measure	Primary Measures Used in Calculation
Alert Bright	No Cue - Neutral Bright
Alert Dim	No Cue - Neutral Dim
Benefit Bright	Neutral Bright - Single Bright Valid
Benefit Dim	Neutral Dim - Single Dim Valid
Cost Bright	Neutral Bright - Single Bright Invalid
Cost Dim	Neutral Dim - Single Dim Invalid
Congruence Benefit	Dual By Dim - Dual by Bright
Dual Asymmetric Cost Bright	Single Bright Valid - Dual by Bright
Dual Asymmetric Cost Dim	Single Dim Valid - Dual by Dim
Dim Better Than Nothing	Single Bright Invalid - Dual by Dim

*Note*. The RT differences between the primary measures in the second column are used to calculate the derived measure in the first column.

### Procedures for Genotyping

Participants produced a saliva sample of approximately 2 ml in an Oragene-250 kit (DNA Oragene, Kanata, Ontario, Canada). DNA sequencing assay was performed to genotype known SNPs (see Table 6). Polymerase chain reaction (PCR) amplifications were carried out using HotStarTaq™ DNA polymerase (Qiagen Inc., Valencia, CA). PCR products were treated using Exo_SAP (Affymetrix, OH) to digest primers and followed with sequencing PCR using the BigDye™ sequencing reaction mix (Applied Biosystems, CA). The sequencing PCR products were purified using the BigDye XTerminator kit (Applied Biosystems, CA) and then loaded on an ABI3730xl sequencing instrument using the Rapid36 run module. The DNA sequencing results were analyzed using the Mutation Surveyor software (SoftGenetics, PA).

**Table 6:** Sequences for Polymorphisms Analyzed.

Polymorphism	Strand	Primer sequence
SL6A3 repeat (*DAT1*)	Sense	5′-TGTGTGCGTGCATGTGG a
	Antisense	5′-GCTTGGGGAAGGAAGGG
rs1108580 (*DBH*)	Sense	5′-ACGCCTGGAGTGACCAGAAG
	Antisense	5′-CCATCCTCCTTGGCTTTCTC
rs429358 (*APOE*)	Sense	5′-GAACTGGAGGAACAACTGAC
	Antisense	5′-CGCTCGCGGATGGCGCTGA
rs7412 (*APOE*)	Sense	5′-GAACTGGAGGAACAACTGAC
	Antisense	5′-CGCTCGCGGATGGCGCTGA
rs4680 (*COMT*)	Sense	5′-GCTACTCAGCTGTGCGCATG
	Antisense	5′-ACGTGGTGTGAACACCTGGT
rs747302 (*DRD4*)	Sense	5′-CGGAGGGAATGGAGGAGGGA
	Antisense	5′-AGACCTGAGCTCAGGCTCTG

*Note.*

a Primer with 5′-Fam fluorescent label.

In the case of the *DAT1* (*SL6A3*) exon 8 polymorphism, genotyping was performed using methods for microsatellite repeat polymorphisms. The fluorescently labeled PCR products were generated with a fluorescently labeled primer (see Table 6). The amplified products were analyzed on an ABI3130xl Genetic Analyzer. The Genemapper 4.0 software was used to assign the allele distribution (Applied Biosystems).

To assess the reliability of the genotyping, we had seven of the participants submit second saliva samples. These samples were treated identically to all of the other samples, and the lab doing the genotyping did not know that they were duplicates of existing saliva samples. The agreement between the two genotyping runs was 97.5% (78 of 80 alleles agreed). Two subjects contributed 10 alleles, and five subjects contributed 12 alleles to the reliability analysis. Each of the two subjects who contributed 10 alleles could not be genotyped on one genetic marker.

### Statistical Analyses

**Figure 6:**
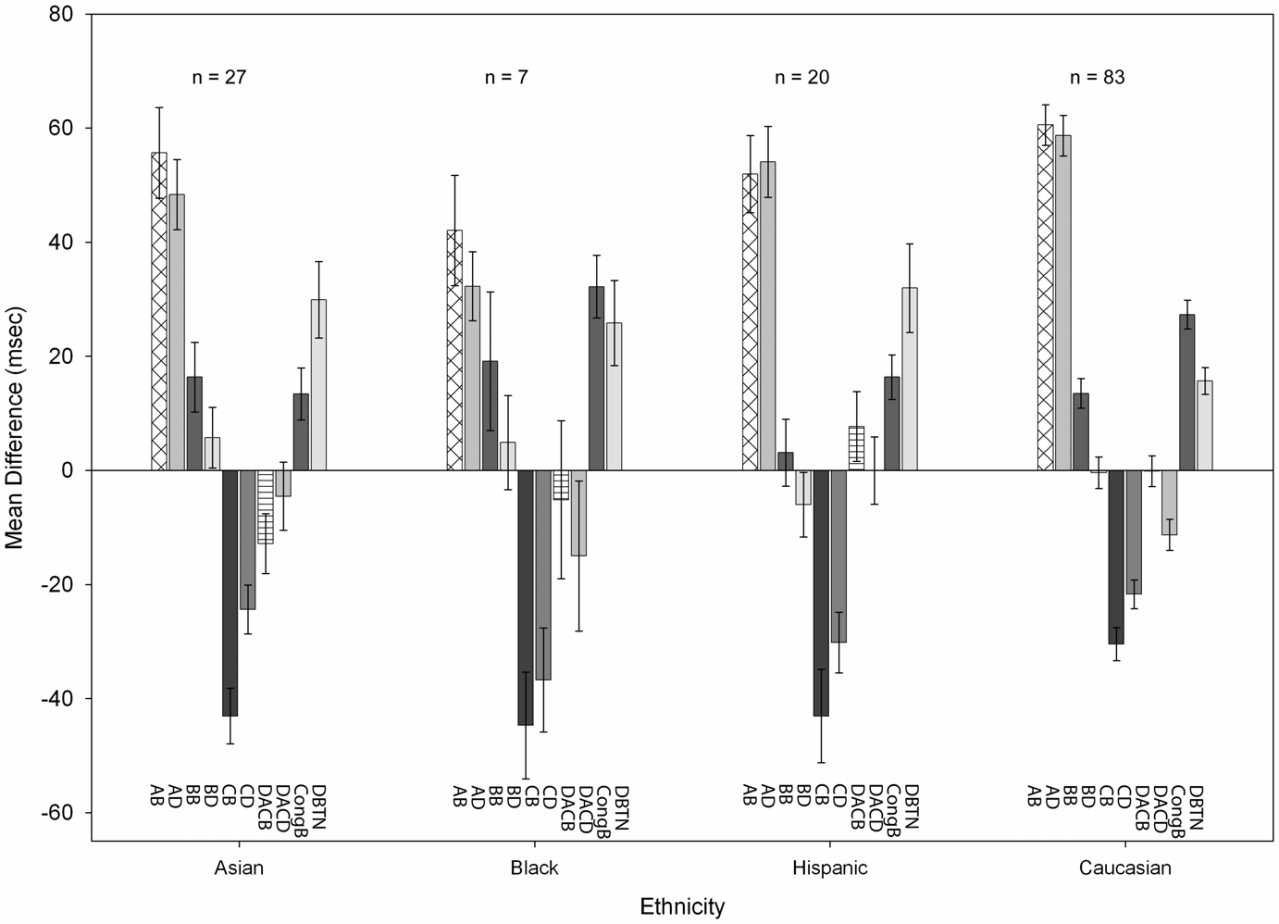
Derived measures by ethnicity. Overall, patterns are similar and statistical decisions were unchanged whether or not ethnicity was used as a factor in the ANOVA. Note that some ethnicities do differ significantly from other ethnicities on particular outcome measures. AB = Alert Bright; AD = Alert Dim; BB = Benefit Bright; BD = Benefit Dim; CB = Cost Bright; CD = Cost dim; DACB = Dual Asymmetric Cost Bright; DACD = Dual Asymmetric Cost dim; CongB = Congruence Benefit; DTBN = Dim Better Than Nothing. Error bars are +/−1 SEM.

We followed the advice of Hutchison, Stallings, McGeary and Bryan [16] to address potential stratification artifacts by using self-reported ethnicity as a proxy for genetic subpopulation. Several of the derived measures showed significant differences by ethnicity (see Figure 6), so we controlled statistically for self-reported ethnicity in all of the genetic associations analyses below. To classify the twelve individuals who reported dual ethnicities, we compared their genetic data to the proportions of those genotypes in our data set and classified each individual into the single ethnicity with which their genetic data were most similar. One individual could not be classified into a single ethnicity in this way, so their data were not used in our analyses.

We followed up the significant MANCOVA (see the Results section) with more focused univariate analyses. For each measure, an initial ANOVA was used with ethnicity as an independent variable to control statistically for possible population stratification artifacts. To test for genetic associations, genotype was then added as another independent variable, and the incremental *R* ^2^ was obtained. Our statistical decisions were unchanged whether or not ethnicity was used as a factor in the ANOAVA. Adding age as a covariate did not change any statistical decisions. We entered genes as predictors in separate models. We did not examine possible epistatic effects (gene×gene interactions) because our sample size was too small to detect these possible effects [51]. In the genetic analyses, the useable sample size of 137 subjects was further reduced because some subjects could not be genotyped at a particular marker. Genotype was entered with *df* = 1 to test for the linear dose/response association (slope) between the number of risk alleles (0, 1, or 2) and the phenotype. This is logical if the increasing dose of a protein produced by an allele leads to progressively more or less neurotransmitter availability. There are studies, however, that suggest other patterns such as dominance effects [35], [52]. We only tested for linear genotype effects because we did not have specific hypotheses about dominance-type effects for any of the markers.
